# Toward just and equitable micro-credentials: an Australian perspective

**DOI:** 10.1186/s41239-022-00332-y

**Published:** 2022-06-01

**Authors:** Renee Desmarchelier, Lisa J. Cary

**Affiliations:** 1 University of Southern Queensland, West St, Toowoomba, QLD 4350 Australia; 2 La Trobe University, Bundoora, VIC 3086 Australia

**Keywords:** Curriculum, Higher education, Micro-credentials, Lifelong learning

## Abstract

The current historic COVID-19 Pandemic moment has thrown into sharp relief the need for flexible and rigorous higher education that meets upskilling and reskilling needs of global workforces. Discussions of micro-credentialing predate the Pandemic but have received increased focus as potentially assisting in addressing perceived skills gaps. However, not all commentators have been complimentary about the possibilities inherent in micro-credentialing. In this paper we discuss Ralston (Postdigital Science and Education 3:83–101, 2021) criticism of the “microcredentialing craze” as provocation to consider how equitable, thoughtful and just educative aims may be met. We address Ralston’s argument that micro-credentials present an educative “moral hazard” by arguing that micro-credentialing will allow universities to respond quickly to changing worker educational needs rather than only offering full degrees that may not be economically viable or personally desirable for individuals. Rather, we suggest, the potential of micro-credentials lies in their pathways and potential to enhance lifelong learning and suggest that micro-credentials do not stand outside of the pedagogical ethical imperative that learning experiences should be positive and inclusive.

## Introduction

Lifelong learning and employability are the cornerstones of the current micro-credential movement in higher education, according to Oliver (2019) and whilst there does not seem to be agreement on ways forward in the sector in Australia, there is certainly interest in the potential for market share. Micro-credentials are seen as a way of meeting upskilling requirements for individuals looking to advance in their career as well as to provide a skilled workforce to rapidly changing industries in an increasingly disrupted world of work (Oliver, 2019). At this historic moment, Australian Higher Education is starved by a lack of funding from the Federal Government, meaning most universities are focused on the competitive student market and are working frantically in crisis mode. Micro-credentials are being touted as an answer to skills shortages with the term entering government discourse around tertiary education (Tehan, 2020). This makes micro-credentialing an attractive diversification. However, we have yet to agree on a definition, let alone move towards a truly collaborative approach on micro-credential offerings (KPMG, 2020; Oliver, 2019; Shapiro, 2020).

There has been work internationally on what a definition of a micro-credential should be with contributions from UNESCO (Oliver, 2021), the European Union (European Commission, 2020) and the development of local frameworks and guides such as in New Zealand (New Zealand Qualifications Authority, n.d.) and Australia (in development). Definitions and explanations to date focus on developing shared understandings of what constitutes micro-credentials to allow for increased portability across educational contexts, better recognition by learners and employers and the need for assessment of learning (Oliver, 2021, Universities Australia, 2021). Common themes in all of this work are that micro-credentials should be assessed, quality assured and offer a transferable, understandable unit of exchange for credit (Desmarchelier, 2021) All of this work indicates the growth and perceived importance to the Higher Education sector internationally but also recognises that micro-credentials are not yet proven in the claims of increasing employability and employment: “Micro-credentials offer exciting possibilities but because it is still early days, the benefits are yet to be realised or proven in many cases” (Oliver, 2021).

In order to consider how the micro-credentialing movement might be situated within the Higher Education sector, we consider the arguments presented by Ralston (2021). We believe it is time to take action, to move beyond issues of definition, and systemic blocks to focus on delivering valuable, worthwhile and useful learning experiences that contribute to lifelong learning.

From our Australian context, the current lack of a supporting national framework and a national system to record credentials and incorporate recognition of prior learning (RPL) and credit makes it almost impossible for potential students and aligned industries to take this learning opportunity seriously (Shapiro, 2020). Applicable across many national contexts, the need for unification in understanding and representation of micro-credentials in a sustainable and futureproof form is recognised as key to meeting the needs of lifelong learners (Shapiro, 2020).

In this piece we focus on the consideration of how micro-credentials can be valuable, worthwhile and useful because:

When the value of different credentials is not clear, students in higher education are discouraged from having their additional learning recognised. Similarly, employers tend not to understand nor be able to assess the quality of alternative micro-credentials as a means to solve their skill demands (MicroHE Consortium, 2019).

### A just and equitable lifelong learning experience

Central to the challenge of developing a ‘good’ micro-credential is defining its value to the learner as well as to the university. While skills development is often framed as a motivation for micro-credential development, this is not the sole consideration for many institutions. Other concerns may be about the public good, providing flexibility and targeted learning, and the cost of education (European Commission, 2020; Oliver, 2021). Implicitly, to achieve value and equity in these areas, learning design, quality assurance and rigor must be appropriate and designed into the course development process. Particular consideration needs to be given to assessment when we are talking about developing a stand-alone lifelong learning experience. Appropriate assessment of learning has emerged as a key factor in attempts to develop shared understandings of microcredentials (Oliver, 2021, Universities Australia). As Oliver (2019) states:

Since assessment is so important to building trust and credibility, micro-credentials could be designed with assessment in mind first and include where possible:

- authentic problem-solving in ill-defined tasks that test real-world application; and
- personal and personalised feedback. (p. 22)

However, Ralston’s (2020) critique of the “micro-credentialing craze” (p.83) raises concerns that universities interests’ seem to be focused on increasing revenue and do not serve the needs of lifelong learning, act in just ways to improve workers’ conditions, or educate the whole person, thus presenting an educational “moral hazard” (p. 96). It is our hope that through establishing the potential value and social contribution of micro-credentialing, the conversation can shift the focus of discussion to just and equitable learning experiences as “the development of micro-credentials needs to be in the service of these big ideas, not as a big idea in itself.” (Brown et al., 2021, p. 250).

We take up the conversation with the backdrop of the development of micro-credentials globally and speculation about the possible negative impacts of the movement. In responding to Ralston’s truth claims, the suggestion that universities are selling out has been previously identified as problematic. Brown et al. (2021) recognised Ralston’s paper as working in “sweeping generalisations” (p. 238) and encourages us to look “beyond the novelty factor, at a deeper level, Ralston (2021) claims that higher education institutions are selling their soul to business interests and market forces by unbundling the degree to quickly bolster their profits” (p. 238). Brown et al. posit that:

Even if one is sympathetic to this line of critique, there is more to the micro-credential story than we have revealed thus far. Importantly, micro-credentials have many different faces and should not be treated or generalised as a single uniform entity. This type of critical stance oversimplifies the micro-credentialing movement… (Brown et al, 2021, p. 238)

So, we have chosen to take up Brown et al.’s challenge and respond to Ralston’s paper from the position of two higher education educators with strong backgrounds in just and equitable educational and previous positioning critiquing neoliberal education. We have focused on identifying where Ralston has made some problematic truth claims in some of his criticisms and where we believe his arguments need to be further considered in order to ensure micro-credentials are valuable, worthwhile and useful.

### Developing a more deliberative, constructive approach to micro-credentialing

Ralston’s (2020) call to arms suggests a “more deliberative and constructive approach to micro-credentialing, an (sic) sound alternative to the current craze” (p. 98). Ralston’s postdigital-Deweyan critique implores readers not to be seduced by neoliberal discourse that frames the sole purpose of education as to get a job (as described by Down, 2009), and avoid what Giroux (2010) describes as a bare pedagogy where education is stripped of its public values and civic responsibility. Ralston promotes a holistic consideration of micro-credentialed education that is not only limited to skills training but also considers enriching the higher education experience through professional identity formation, cultivation of soft skills and the ability for a student to appreciate “the extent of their own ignorance” (p. 97). Ralston’s proposed postdigital alternative to the micro-credentialing craze goes beyond a focus on hard or technical skills and recognises an obligation to “uplift learners, liberating their potentiality and empowering them to become ‘masters of their own industrial fate’” (p. 94, Ralston citing Dewey). Indeed, such calls for a socially responsive and responsible agenda are congruent with our own views of our responsibilities as educators in evolving micro-credential spaces.

Ralston’s detailed postdigital-Dewean critique identifies ten issues with micro-credentialing. We find ourselves in agreement with elements of the issues raised but consider further discussion and debate as essential to moving towards valuable, worthwhile and useful micro-credential experiences. In the first issue Ralston identifies speaks to the potential negative impacts of a narrowly focused skills agenda in higher education:

Micro-credentialing is dangerously reductivist. It reduces higher learning to a list of hard skills and technical competencies that bolster employer workforce development and heighten employees’ earning potential. Soft skills and human competencies to, for instance, ‘learn to learn’ are arbitrarily excluded from micro-credential curricula, even though they contribute to career development and personal growth (Wilhelm et al., 2002; Grugulis & Vincent, 2009). (Ralston, 2020, p. 95)

The international micro-credential landscape is a multidimensional hotch-potch of credentials, providers, and platforms. The type of organisation or institution producing the micro-credential has a profound impact on the positioning of educative aims for micro-credentials (Desmarchelier, 2021). Writing from the Australian context, many universities are active in the micro-credential space and offerings are many and varied but usually cater for learning beyond skills development only. In some cases, soft skills like teamwork, critical thinking and digital literacy are the credentials on offer (see Deakin University, 2021). Professional identity development is the focus of other micro-credentials where the learning approach is designed for professionals in the workplace recognising “this stage of professional learning is about transformation, the application of your professional identity and consideration of how to apply specialist and technical knowledge to contexts and contingencies” (USQ UpSkill, 2020).

While some technical education focused on skills development is seen in the diverse field of international offerings, many institutions, particularly universities, often recognise and implement micro-credentials that make contributions to an individual’s holistic professional development as core to the educative experience. If we take Brown et al.’s (2021) categorisation of micro-credentials as “unbundled, credit-bearing, stackable credentials” (p. 232) it necessarily follows that there needs to be clear, mapped pathways for credit into macro-credentials (e.g., degrees). For stacked micro-credentials to be recognised as credit, learners must meet equivalent learning outcomes as they would if they had completed the full units in the degree program. This is not to say that learning outcomes will necessarily be identical, there may be more than one way for a student to demonstrate attainment of the required theory, knowledge, and skills to meet degree-level requirements. While one micro-credential on its own is not going to deeply develop professional identity and ‘learning to learn’ proclivities, we could foresee that the opportunity to flexibly delve in and out of micro-credentials (and stack to full degrees) through lifelong learning approaches will have this benefit for learners. Of course, to achieve these aims, micro-credentials must display rigor and quality assured learning representative of a particular level of study.

Ralston also asserts, “Micro-credentialing contributes to the decline of the traditional degree. It paves the way for the total substitution of degree programs with micro-credentials…” (p. 95). To this issue, we would ask, what is sacred about a traditional degree structure? We see the undergraduate/postgraduate degree structure as firmly embedded globally in neoliberal education systems that require the expenditure of (usually large) amounts of money from varying mixtures of private individual and public government sources. We acknowledge the legitimacy of Ralston’s commentary about how universities are looking to profit from offering micro-credentials and the potential economic advantages to a university of unbundling and servitization as possibly negatively impacting quality higher education. However, we would also ask if concerns related to the impact of neoliberalism on higher education are absent from, or less prominent in, many traditional degree programs. Universities are increasingly beholden to economic imperatives and efficient delivery of learning is a goal at most institutions. At its best, micro-credentialing may provide a different model of lifelong learning that is more flexible and better meets the needs of learners to become ‘masters of their own industrial fate’ (Dewey cited by Ralston, 2020). Increasingly, public media is questioning the worth of traditional degrees and examination of the cost of a degree versus the economic gains from its study are widely discussed (see Benedict et al., 2021 as an example). There is building evidence from the United States of America that forms of non-degree credentials, for example educational certificates and professional certifications, lead to participants’ believing they are a more attractive job candidate, consider the education to be worth the cost and are well valued by Black Americans as helping achieve educational goals (Strada Centre for Education Consumer Insights, 2021). However, at this time, it is recognised that there is a dearth of in-depth data an empirical analysis in regard to the tangible individual benefits of micro-credentials (Brown & Mhichil, 2021).

Related to the value of a traditional degree, Ralston also considers micro-credentialing as undermining of the mission of higher education and a “moral hazard” (p. 95) of profitization and commodification of the process of learning. Regarding commodification and profitization, we argue that higher education, as a neoliberal system, has engaged in these activities for many years. Often, making profit from one source can cross-subsidise education and research in other areas within an institution. In Australia, the global nature of the COVID-19 pandemic has thrown into sharp relief how reliant our higher education sector has been on income from international students. Often, international student income provided funding for research and other institutional activities. Widespread job losses and threats to research funding resulted from the closure of Australia’s international borders. Many degrees in Australia offer Commonwealth supported places for Australian residents where the government covers part of the cost of a student’s study and the student has the option to defer payment of the remainder of the cost until they are in paid employment. Currently, micro-credentials (stackable for credit sitting outside of degree structures) do not attract Commonwealth funding support and so may be seen as generating additional revenue for universities. As such, micro-credentials do become an attractive addition to a university’s learning options during such a time of global uncertainty and diminished balance sheets. The COVID-19 pandemic has accelerated the need for workers to up-skill and re-skill (Brown, 2021). Rather than presenting a “moral hazard” we would argue that micro-credentialing will allow universities to respond quickly to changing worker educational needs rather than only offering full degrees that may not be economically viable or personally desirable for individuals. Indeed, one of the cited motivations for developing the micro-credential marketplace in Australia is to address “the most common barriers cited by adult workers who are not intending to undertake further formal training or study: time and cost” (Tehan & Cash, 2020).

Ralston highlights that neoliberal economic burdens mean that universities are increasingly under pressure to deliver efficient models of education that are market competitive. Here we have no argument with Ralston. Even in Australia where education is more socialised, students from lower socio-economic status backgrounds, first in family students and mature learners with a family to support (and many others) often struggle with the financial commitment to be able to study either in the form of direct cost (albiet deferred) or the opportunity cost of time out of the workforce (Lamb et al., 2020). Where our position differs to Ralston is in our understanding of the potential of micro-credentials to offer an alternative to full degree programs that may assist in upskilling and therefore finding more meaningful and lucrative participation in the workforce. In addition, there seems to be some thought that learners may see micro-credentials as ‘dipping their toes in the water’ for further traditional higher education study with many universities promoting microcredentials as stackable for credit to provide pathways into macro qualifications. The potential to “exacerbate class divisions” (p. 97) could be inferred if workers can only afford to access micro-credentials rather than to full degrees. We argue that if integrated national frameworks assuring learning quality and level are developed allowing shared understanding of the value of micro-credentials, the economy will evolve a different conception of qualifications. As a result, in some areas the value of a traditional degree may be viewed differently, potentially mitigating some of this concern.

Ralston is not alone in his criticism of unbundling current courses to develop ‘new’ micro-credentials:

Rather than presenting new opportunities for social inclusion and access to education, they [micro-credentials] contribute to the privatisation of education by unbundling the curriculum and blurring the line between public and private provision in higher education (Wheelahan & Moodie, 2021, p. 1)

There is also some concern within Australian higher education that it is not possible to simply incorporate micro-credentials into existing course units, and that the current practice of unbundling and repackaging existing course units into micro-credentials is unsound (Boud & Jorre de St Jorre, 2021). A consideration when unbundling learning occurs is the maintenance of rigor and quality and the cohesiveness of the learning offered. To produce quality assured micro-credentials from existing material requires significant development input meaning the cost of developing micro-credentials is often significant. While Ralston rightly points out that unbundling of degree content can be the source of materials for micro-credentials, developing meaningful learning experiences that contribute to professional identity and ‘learning to learn’ agendas requires skillful and intentional application of learning design and pedagogical principles. Applying clever and clear learning design approaches that prioritise soft skill and professional identity development through embedded learning design means that liberation of potentiality of learners may not be completely compromised. Where attention to such quality issues is observed, institutions may find that the unbundling process requires specialist teams of educators and directed financial investment, which is the other side of the coin to Ralston’s neoliberal argument.

As Brown et al. (2020) point out, part of Ralston’s argument hinges on the perceived challenge to higher education as a public good. During the COVID-19 pandemic, micro-credentialing has demonstrated its potential to contribute to public good in several ways. The flexibility to develop and offer this type of learning meant it could be quickly leveraged to provide up-to-date knowledge and skills for workers to increase their employability in a time of economic uncertainty. In some arenas, micro-credentialing has been at the heart of COVID-19 responses and contributed to providing safer workplaces. One example in Australia was the quick repurposing of micro-credentialing to deliver required competencies in COVID-safe practices in industries like hospitality and healthcare (Queensland Government, 2021). Arguably, these micro-credentials have contributed to protecting many lives and livelihoods, including the participants in the learning and the members of the public they serve. In addition, many institutions have offered previously fee-attracting micro-credentials as free upskilling for participants to contribute to their community’s economic recovery (Toowoomba Chronicle, 2020).We have seen the quick flip of agendas from increasing revenue to contributing to the public good from several universities and vocational education intuitions in Australia.

Ralston’s fifth issue addresses concern that “microcredentialing does not liberate learners’ potentialities or meet the needs of lifelong learners” (p. 96). The assumption underlying this issue is that “microcredentials pertain exclusively to the acquisition of industry-specific skills and competencies, they do not cultivate habits of intelligent inquiry that are fundamental to intellectual curiosity and self-exploration” (p. 96). Again, Ralston brings into focus Dewey’s notion of learning-to-learn rather than learning to earn. As we have argued already, we reject the generalised assumption that micro-credentials only pertain to industry-specific skills and competencies and have demonstrated how universities are responding in ways that develop learning-to-learn proclivities. One point that requires further discussion is that while content in micro-credentials may pertain to development of professional identity and lifelong learning skills, to ensure learners acquire these desired learning outcomes requires that they are appropriately assessed. It is not enough to embed lifelong learning in content, appropriate rigor and quality assurance of stated learning approaches mean it must be part of assessment as well.

In addition to lifelong learning, the potential of life-wide learning via micro-credentials needs to be considered in relation to Ralston’s arguments. Life-wide learning allows individuals to “meet their personal goals and help foster broader social outcomes such as general health, social trust, political efficacy and civic engagement” (Reder, 2020). Individuals may invest in micro-credentialed education to improve their numeracy or literacy skills, better understand and improve their and their family’s health and wellbeing, satisfy aspirations like writing a book, or to be able to better participate in activism and democratic processes. As such, micro-credentialing may contribute to life-wide learning that potentially liberates individuals and communities while meeting the needs of universities for diversified income sources.

In moving towards a shared understanding of micro-credentials, Oliver (2021) in the UNESCO Draft Preliminary Report highlights the possible contribution of the movement to achieving quality education:

there is strong hope that micro-credentials can advance the equity agenda, bringing accessible and affordable focused learning and skill building to vulnerable communities, enabling achievement of the United Nations Sustainable Development Goal 4 (Quality education) (Oliver, 2021)

Ralston’s concern that micro-credentials do not liberate learners does not seemed to be shared by Oliver (2021). Specifically, we suggest, micro-credentials could assist in moving towards the following Sustainable Development Goals:

- 4.3 Equal access to technical/vocational and higher education and
- 4.4 Relevant skills for decent work.

While the basis of Ralston’s first five arguments relates to learning-to-earn vs learning-to-learn, issues six to nine focus on a Marxist analysis that micro-credentials will not challenge the status quo and improve workers’ lives, potentially exacerbating class divides. These points are considered together as their substance relies on the same argument and they repeat some of the earlier concerns tying them to a Marxist analysis. Ralston argues “If inequality between owners of capital and workers, who solely own and sell their labor is to end, micro-credentialing will not bring it about instead bonafide social change requires a more holistic approach to higher education” (p. 96). To this we agree that neither micro-credentialing nor traditional higher education is formulated to disrupt the status quo. However, making access to tertiary education more available at lower price points, either through discounted introductory options or the ability to study only parts of whole degrees, may inch towards increasing equity in participation. Rather than forcing potential students into lengthy expensive degrees, micro-credentialed offerings that can be accessed as either lifelong or life-wide learning needs arise mean more and cheaper access to education than previously available.

The last issue of concern, in Ralston’s estimation is the unjustifiable privilege of digital over non-digital or pre-digital technologies. We argue that in current times, it is not simple to untangle which modes of delivery should be privileged. There is much debate currently around what the teaching environments of Australian universities should look like in a post-COVID world. Some argue that students do not want an end to the face-to-face traditional lecture delivery model (Vanderberg & Cowling, 2021). Others argue that delivery should be and is becoming more contextualised to the particular types of student and institution resulting in various levels of face-to-face, online and blended (incorporating both) learning (Sankey & Campbell, 2021) Where higher education institutions in Australia already had good online learning capabilities, the COVID disruption to students was less than in situations where face-to-face learning had been privileged. In the case of micro-credentials, the digital allows for global access to education in a way never seen before. A course can be offered by an Australian university and have participants from South America, Asia and Europe, making for an enriched learning environment for students. It must also be recognised that not all micro-credentials are offered as online only learning, in the Australian context at least, some face-to-face opportunities to earn micro-credentials exist.

### A final word—what now?

While much of our writing could be read as critical of Ralston’s position, we are sympathetic to the call to arms to enact micro-credentialing in equitable, thoughtful and just educative ways. Internationally, calls for attention to the skills agenda are apparent in high level reporting from government and private sector commentators looking at jobs markets. Such perspectives could be challenging for academics who have perceptions rooted in more traditional concepts of a university education. The challenge we see is to harness the momentum created by calls to consider how micro-credentials can advance the equity agenda and meet United Nations sustainable development goals.

Micro-credentialing represents a potential seismic shift in the global landscape of higher education. Most institutions will have pockets of highly innovative learning and teaching practice driven by committed academic staff. To make micro-credentialing successful, these need to be harnessed and directed at a whole of institution level. We would suggest this direction needs to encompass not only quality assured learning and teaching but include Ralston’s concerns, consideration of equitable, thoughtful, and just educative approaches to micro-credentialing. Evidence around the successful implementation of educational innovation suggests the need for a clear whole of institution approach with Lašákováa, et al. (2017) suggesting:

…it is important to facilitate cooperation among colleagues, to stay open to new ideas, to share power, and dedicate time for teamwork (Garcia and Roblin, 2008). Furthermore, as Garrison and Kanuka (2004) found out in their study on successful adoption of a new bended learning approach, the creation of clear institutional policies for innovation, establishment of supportive organisational structures at HEIs, such as contact points or specialised units, and a managerial strategic approach to innovation selection and evaluation, are essential.”

While such measures may or may not satisfy Ralston’s critique of the micro-credentialing craze, to address individual concerns such as those raised by Ralston, a thoughtful and strategic approach is necessary. Points raised by Brown et al (2021) need to be considered in the development of strategic approaches and the advancement of micro-credentialing internationally:

Ultimately, we need longitudinal research to establish whether our investment in micro-credentials has contributed to significant private and public benefits. Currently, there is scant evidence to show whether micro-credentials are helping to address skill gaps, develop closer partnerships between universities and industries, and promote new educational pathways that increase the level of participation in lifelong learning. (pp. 249–250)

## Conclusion

Brown et al. (2020) remind us that this discussion needs to be about more than just a ‘big idea’. Micro-credentials do not stand outside of the pedagogical ethical imperative that learning experiences should be positive and inclusive (Bozkurt et al., 2020). Within this current context we should take note of Ralston’s (2020) concern that the sector should be about more than skill development. Knowledge, skills and values should all be considered part of the micro-credential curriculum (Cary, 2006), and the learning experience needs to be grounded in good academic work that is owned by the teaching staff.

The development of the micro-credentialed curriculum should be led by staff with skills in leadership in learning and teaching, and not only the ‘business’ partners within the institution. Building these relationships takes skill and time, something we do not have a lot of at the moment, and the sense of urgency is in danger of derailing the reform. However, the current COVID context is an historic moment that centres the importance of learning and teaching in higher education.

Whilst understanding the requirement for agile systems (within and across institutions) and econo-centric concerns, we believe that the need to start with a grounded approach. If we move forward into the space of possibility, where internal and external systems suddenly exist, we should also consider the re-envisioning of higher education as part of the social contract of democratic education:

Let us therefore take this opportunity to redefine education and learning as a single, continuous, flexible pathway from birth to death that should be facilitated and funded properly and fairly, whether by the taxpayer, the student, endowments, or employers or in a combination of ways, and remove stigmas attached to level or type of study, the name or type of provider, or affordability. (Kirkham, 2021, p. 262).

This reform holds the promise of access to lifelong learning and so we call for attention to the fact that it is “not just the content of the curriculum that matters” (Wheelahan & Moodie, 2021, p. 223). Wheelahan and Moodie (2021) remind us that the micro-credential reform is also contributing to changing the very nature of higher education curriculum and it is this aspect that we believe must be considered.

## Data Availability

Not applicable.
